# Title Screening Utility of the Mood Disorder Questionnaire (MDQ) for Detecting Bipolar Disorder in the Perinatal Period: A Pooled Analysis

**DOI:** 10.1192/j.eurpsy.2026.11947

**Published:** 2026-07-31

**Authors:** B. Larisa, I. Nastas

**Affiliations:** Department of Mental Health, Medical Psychology and Psychotherapy, USMF, Chisinau, Moldova, Republic of

## Abstract

**Introduction:**

Bipolar disorder during pregnancy and the postpartum period is frequently under-recognized because physiological and psychosocial perinatal changes (e.g., sleep loss, variability in energy and mood) can mimic hypomanic symptoms. Failure to identify the disorder is associated with relapse, antidepressant-induced switching, and adverse maternal–infant outcomes. Although the Mood Disorder Questionnaire (MDQ) is widely used, its standalone screening performance, optimal thresholds, and the balance between sensitivity and specificity in perinatal care remain uncertain due to heterogeneous scoring rules across studies.

**Objectives:**

To synthesize the diagnostic performance of the standalone MDQ in perinatal populations, characterize the sensitivity–specificity trade-off and heterogeneity, and illustrate discriminative performance in an independent sample.

**Methods:**

We searched MEDLINE/PubMed and Google Scholar (2011–2023) for studies of perinatal women using the MDQ as a standalone screening tool, with bipolar disorder verified by SCID or MINI and sensitivity/specificity reported. Six studies met criteria, yielding 15 sensitivity–specificity pairs. We computed Pearson’s, Spearman’s, and Kendall’s correlations, tested normality (Shapiro–Wilk), and assessed heterogeneity (Cochran’s Q, I²). ROC–AUC was estimated in an independent subsample (n = 30).

**Results:**

Mean sensitivity was 83.9% and specificity 78.5%. Sensitivity and specificity showed a negative monotonic association (ρ (Spearman) = −0.57; τ (Kendall) = −0.50; both p < 0.05). Distributions were asymmetric and non-normal. Heterogeneity was moderate to substantial (χ²(14) = 34.39; I² ≈ 59%). No significant differences were found between pregnancy and postpartum subgroups (ANOVA: F(1,28) = 1.75; p = 0.197; Mann–Whitney: U = 105; p = 0.771). In the independent subsample, discriminative ability was low (AUC = 0.544; 95% CI includes 0.5).

**Conclusions:**

In perinatal care, the MDQ shows acceptable sensitivity with moderate specificity and a clear trade-off, supporting its use as a screening tool. Positive screens should be confirmed via structured clinical interview and, where appropriate, complemented by perinatal scales. Threshold selection should be adapted to clinical context, expected prevalence, and the acceptable balance of false negatives and false positives. Limitations include the small number of standalone MDQ studies, reliance on aggregated metrics rather than individual-patient data, and potential publication bias.

**Disclosure of Interest:**

None Declared

